# Heat exposure limits pentose phosphate pathway activity in bumblebees

**DOI:** 10.1093/conphys/coae031

**Published:** 2024-05-28

**Authors:** Giulia S Rossi, Alaa Elbassiouny, Jerrica Jamison, Kenneth C Welch Jr.

**Affiliations:** Department of Biological Sciences, University of Toronto, 1265 Military Trail, Scarborough, ON M1C 1A4, Canada; Department of Biology, McMaster University, 1280 Main St W, Hamilton, ON L8S 4E8, Canada; Department of Biological Sciences, University of Toronto, 1265 Military Trail, Scarborough, ON M1C 1A4, Canada; Department of Biological Sciences, University of Toronto, 1265 Military Trail, Scarborough, ON M1C 1A4, Canada; Department of Biological Sciences, University of Toronto, 1265 Military Trail, Scarborough, ON M1C 1A4, Canada

**Keywords:** Climate change, glucose, metabolic pathways, oxidative stress, pollinators, thermal stress

## Abstract

Bumblebee populations across the globe are experiencing substantial declines due to climate change, with major consequences for pollination services in both natural and agricultural settings. Using an economically important species, *Bombus impatiens*, we explored the physiological mechanisms that may cause susceptibility to extreme heat events. We tested the hypothesis that heat exposure limits the activity of the pentose phosphate pathway (PPP)—a parallel pathway to glycolysis that can use nectar sugar to generate antioxidant potential and combat oxidative stress. Using isotopically labelled glucose, we tracked PPP activity in *B. impatiens* at rest, during exercise and during a post-exercise recovery period under two different temperature regimes (22°C and 32°C). We found that the PPP is routinely used by *B. impatiens* at moderate temperatures, but that its activity is markedly reduced when ATP demands are high, such as during periods of exercise and heat exposure. We also exposed *B. impatiens* to either 22°C or 32°C for 5 hours and assessed levels of oxidative damage (lipid peroxidation, protein carbonyls) and antioxidant potential [reduced (GSH) and oxidized (GSSG) glutathione concentrations]. Interestingly, bees exhibited little oxidative damage after the thermal exposure, but we found a lower GSH:GSSG ratio in 32°C-exposed bees, reflecting lower antioxidant potential. Overall, our study demonstrates that acute heat stress severely limits PPP activity and may constrain antioxidant potential in *B. impatiens*. The repeated attenuation of this pathway in a warming climate may have more severe physiological consequences for this species, with potential implications for pollination services across North America.

## Teaser

Bumblebee declines threaten pollination services worldwide. We explored the physiological mechanisms that may explain why bumblebees are susceptible to heat stress. We discovered that elevated temperatures limit the activity of the pentose phosphate pathway—a parallel pathway to glycolysis that generates antioxidant potential crucial for mitigating oxidative stress.

## Introduction

Bumblebees (*Bombus* spp.) provide vital economic and ecological benefits by enhancing crop yields (Klein *et al.,* 2007; Ricketts *et al.,* 2008) and supporting wild plant populations through pollination (Aguilar *et al.,* 2006; Ollerton *et al.,* 2011). Their value as pollinators is often attributed to their generalist foraging strategy and their capacity to pollinate at colder temperatures than many other insects (Goulson, 2003). Moreover, bumblebees are capable of ‘buzz pollination’, in which thoracic muscle vibrations dislodge pollen that is firmly held by flower anthers (De Luca and Vallejo-Marín, 2013). Many economically valuable crops (e.g. tomatoes, blueberries, pumpkins, zucchini, cranberries) depend on buzz pollinators for successful pollination. Consequently, certain bumblebee species—primarily *Bombus impatiens* from North America and *Bombus terrestris* from Eurasia—are cultivated to provide pollination services for agriculture, contributing billions of dollars annually to crop production worldwide (Velthuis and Van Doorn, 2006).

Both wild and cultivated bumblebees are threatened by climate change, with extreme heat events being particularly detrimental to this cold-adapted genus (Maebe *et al.,* 2021). Indeed, elevated temperatures were found to reduce bumblebee foraging behaviour (Descamps *et al.,* 2021; Gérard *et al.,* 2022; White and Dillon, 2023), flight performance (Kenna *et al.,* 2021; Kuo *et al.,* 2023), as well as learning and memory (Gérard *et al.,* 2022). At the population level, several bumblebee species have suffered substantial declines in recent years (Graves *et al.,* 2020; Soroye *et al.,* 2020). For example, *Bombus occidentalis* was once considered one of the most widespread bumblebee species in western Canada but has now been extirpated from several regions in the southern (warmest) part of its range (COSEWIC, 2014). In contrast, *B. impatiens* has expanded its range across North America and increased in abundance despite a warming climate (Colla and Packer, 2008; Grixti *et al.,* 2009; Looney *et al.,* 2019). Given the growing contribution of *B. impatiens* to pollination services in both natural and agricultural settings, understanding the physiological responses of this species to extreme heat events has become an important area of investigation.

Like all other bumblebees, *B. impatiens* feed largely on nectar, which is composed of sugars that are critical for generating the energy (ATP) required for powering virtually all physiological processes. A well-established consequence of aerobic ATP production is the generation of reactive oxygen species (ROS), which may cause oxidative damage to nucleic acids, proteins and lipids if produced in excess (for reviews, see Storey, 1996; Pamplona and Costantini, 2011). At elevated temperatures, physiological processes operate at a faster pace, thereby increasing ATP demands and potentially ROS production. Thus, bumblebees may be particularly susceptible to oxidative damage during extreme heat events associated with global climate change. Moreover, intense exercise (e.g. flight and buzz pollination) will also increase ATP demands and produce ROS that can cause damage to the flight muscles (Levin *et al.,* 2017; Guo *et al.,* 2020). Animals can mitigate oxidative damage using antioxidants produced endogenously or obtained though dietary sources (e.g. berries, fruits, nuts) to scavenge and neutralize ROS. However, floral nectar is generally low in antioxidants (Baker, 1977), which may constrain the capacity of *B. impatiens* to combat oxidative damage and/or select for enhanced capacities for endogenous antioxidant production, such as the upregulation of various genes (e.g. thioredoxin) linked to antioxidant defence (Choi *et al.,* 2006; Li *et al.,* 2020).

Levin *et al.* (2017) recently hypothesized that nectivores may mitigate oxidative damage by shunting dietary sugar to the pentose phosphate pathway (PPP)—a parallel pathway to glycolysis that can generate antioxidant potential. During the first step of glycolysis, glucose is converted to glucose-6-phosphate (G-6-P). A portion of this G-6-P can be shunted away from the glycolytic pathway to the PPP where it is oxidized and decarboxylated, generating ribulose-5-phosphate and NADPH (Stincone *et al.,* 2015; Cho *et al.*, 2018). Ribulose-5-phosphate is an important building block for nucleic and amino acids, whereas NADPH plays a critical role in recycling glutathione, an essential antioxidant in its reduced form (GSH) against reactive oxygen and nitrogen species (Jozefczak *et al.,* 2012; Vašková *et al.,* 2023). Indeed, fed nectivorous hawkmoths (*Manduca sexta*) were found to shunt nectar glucose to the PPP during periods of inactivity, resulting in lower oxidative damage to flight muscles during subsequent flight activity (Levin *et al.,* 2017). The use of the PPP may require that insects partition the metabolism of sugar to balance the need for ribose-5-phosphate and NADPH with the need for immediate ATP production (Thompson, 1999). When energetic demands are elevated above those during resting conditions (e.g. during extreme heat events and bouts of exercise), do insects limit PPP activity in favour of immediate ATP production? If so, reduced PPP activity may have consequences for a number of life-history characteristics given that ribulose-5-phosphate is important for growth (Jiang *et al.,* 2014) and that NADPH can help combat oxidative damage, a major mediator of longevity (Liguori *et al.,* 2018; Li-Byarlay and Cleare, 2020). Thus, understanding how elevated temperatures alter PPP activity may be critical for predicting the susceptibility of animals to oxidative damage in a warming world.

Here, we tested the hypothesis that *B. impatiens* utilizes the PPP to generate antioxidant potential and mitigate oxidative damage, except during periods of intense exercise and/or heat stress. Using carbon position-specific isotopically labelled glucose, we tracked PPP activity in *B. impatiens* at rest, during exercise and during a post-exercise recovery period under two different temperature regimes (22°C and 32°C). We predicted that bees exposed to 32°C would exhibit lower PPP activity than bees exposed to 22°C given the higher ATP demands associated with elevated temperatures. Moreover, we predicted that all bees, irrespective of the experimental temperature, would show reduced PPP activity during periods of exercise (e.g. flight). Finally, we exposed an additional group of bees to either 22°C or 32°C for several hours to assess markers of oxidative damage (i.e. lipid peroxidation, protein carbonylation) and antioxidant potential (i.e. [GSH], [GSSG] and GSH:GSSG). The goal of this additional thermal exposure experiment was to mimic any temperature-related changes in PPP activity and determine whether these changes in PPP activity would correlate with oxidative stress and antioxidant defence markers, given the role of the PPP in generating antioxidant potential and combating oxidative stress.

## Materials and Methods

### Experimental animals

We captured 29 female bumblebees (*B. impatiens*) that were foraging on *Salvia* spp. in Toronto, Ontario, Canada, at the University of Toronto Scarborough (0.21 ± 0.01 g). All bumblebees were captured between 23 June 2022 and 19 August 2022, between 7:00 AM and 2:00 PM. Upon capture, the bumblebees were transported to the laboratory (room temperature, 22°C) and placed within a large cylindrical Plexiglass container (8 l) for up to 1 hour to recover from handling stress. Each bee was provided with 150 μl of 25% sucrose solution during this recovery time, enriched with 1 mg ml^−1^ of ^13^C glucose (Cambridge Isotope Laboratories, Inc.). A subset of bumblebees were fed glucose labelled on carbon number 1 (^13^C_1_) (*n* = 14), and the remaining bees were fed glucose labelled on carbon number 2 (^13^C_2_) (*n* = 15). The sucrose solution was placed on a piece of Parafilm (Bemis) in three droplets of 50 μl each. We estimated the amount of sucrose solution ingested by the bumblebees by carefully pipetting away the remaining solution (to the nearest 10 μl) after they had retreated heir proboscis and moved away from the droplets. There was no difference in the amount of sucrose solution consumed between ^13^C_1_- and ^13^C_2_-fed bees (Supplementary Fig. S1). Immediately after feeding, bumblebees were placed in a cylindrical glass respirometry chamber (200 ml) to assess the fate of either ^13^C_1_ or ^13^C_2_ during periods of rest, exercise and post-exercise recovery. All six carbons on the glucose ring (C_1–6_) are normally passed through glycolysis and are oxidized in the mitochondria as acetyl coenzyme A (acetyl-CoA). However, when glucose is shunted through the PPP, C_1_ is removed during the oxidative phase of the PPP and leaves as CO_2_ in the breath. Thus, a greater proportion of ^13^C_1_ relative to ^13^C_2_ (or ^13^C_3–6_) in exhaled CO_2_ would be indicative of greater PPP activity (Levin *et al.,* 2017).

To assess oxidative damage and antioxidant potential, we captured an additional 32 female bumblebees foraging on *Rosa rugosa* at University of Guelph, Ontario, Canada (0.20 ± 0.05 g). All bumblebees were captured on 5 July and 6 July 2023 between 9:00 and 9:45 AM. Shortly after capture, the bees were individually placed in plastic petri dishes (9 cm in diameter, 1.5 cm tall; Fisherbrand™ 431 761) containing either 200 μl of a 25% sucrose solution (*n* = 16) or 200 μl of dechlorinated tap water (*n* = 16) in small bowls made of Parafilm. Our rationale for offering a subset of bumblebees with a 25% sucrose solution was to provide access to nectar glucose that could be utilized by the PPP. We offered the remaining bees water to minimize evaporative water loss differences between groups and maintain humidity in petri dishes at similar levels. From each group, half of the bumblebees were exposed to 22°C (*n* = 8 per dietary treatment) and half to 32°C (*n* = 8 per dietary treatment) for 5 hours in an incubator (Innova 4230, New Brunswick Scientific), although one bee provided with water at 32°C did not survive the exposure and was excluded from all analyses. We chose a 5-hour period because it reflects the approximate duration of the respirometry experiments and provides an ecologically relevant exposure to peak summer temperatures (e.g. 11 AM to 4:00 PM). Throughout the 5-hour exposure, we video recorded the bumblebees to assess voluntary walking activity using a webcam (Ausdom Web Camera, HD 1080P) and determined the total distance travelled from videos using EthoVision XT software (Noldus). Flight was not possible in the petri dishes given their ~ 1.5-cm height. Immediately following the exposure period, bumblebees were sacrificed by flash freezing in liquid nitrogen and maintained at −80°C until biochemical analyses were performed.

### Stable isotope tracking and respirometry protocol

After placing bumblebees into respirometry chambers, we allocated a subset of ^13^C_1_- and ^13^C_2_-fed bumblebees to one of two ecologically relevant summer temperature treatments: 22°C (^13^C_1_-fed = 7; ^13^C_2_-fed = 7) and 32°C (^13^C_1_-fed = 7; ^13^C_2_-fed = 8). The high temperature exposure was achieved by placing the respirometry chamber approximately 20 cm below a heat lamp. The chamber warmed at approximately 1°C per minute, as it took approximately 10 minutes for the respirometry chamber to reach a stable 32°C from room temperature. We monitored the temperature within the chamber throughout the experiment using a digital thermometer (Zacro). For both temperature groups, CO_2_-free air was passed through respirometry chambers at a flow rate of 150 ml min^−1^ using a mass flow controller (Sable Systems International). A subsample of 30 ml min^−1^ was pulled from an excurrent manifold directly into a gas analyser (Turbofox), which took CO_2_ and water vapour measurements every second. An additional subsample of 50 ml min^−1^ was pulled from the manifold into a stable carbon isotope analyser (Picarro, Santa Clara, CA, USA) to assess the δ^13^C signature in the breath.

We covered the respirometry chambers with a dark cloth to encourage the bumblebees to cease activity and reach a resting state. In the 22°C group, we deemed bumblebees to be at rest when intermittent ventilation was exhibited (i.e. briefs periods of ‘breath-holding’ followed by bursts of CO_2_ release). Because bumblebees in the 32°C group never exhibited intermittent ventilation, we deemed these bees to be at rest when CO_2_ production rates reached a low, steady state. In both temperature groups, rest was achieved in the covered chamber after approximately 2–4 hours in the chamber. Following the rest period, we prompted bumblebees to exercise for 3 minutes by removing the dark cloth and shaking the chamber gently to stimulate activity. When the bees were disturbed from rest, they activated their flight muscles in a pre-flight warm-up and exhibited some flight within the chamber. After the 3-minute exercise period, we returned the bumblebees to the dark to allow for recovery. Recovery measurements were obtained when the 22°C group resumed intermittent ventilation and the 32°C group exhibited steady-state CO_2_ release (after approximately 30–60 minutes in both groups). All bumblebees were weighed and released back to their place of capture when the experiment was terminated.

### Biochemical assays

To assess lipid peroxidation, protein carbonyl content, reduced (GSH) and oxidized (GSSG) concentrations and the GSH:GSSG ratio, we homogenized frozen bumblebees in 10× volume of ice-cold homogenization buffer (50 mM potassium phosphate, 1 mM ethylenediaminetetraacetic acid (EDTA), 0.1% Triton X-100; pH 7.4). We centrifuged the homogenates for 5 minutes at 20 000 G (4°C) and used the supernatant in all biochemical assays after storage at −80°C. We determined the protein concentration in the supernatant of each sample using bovine serum albumin as a standard and standardized all results accordingly.

Lipid peroxidation was assessed using the QuantiChrom™ TBARS assay kit (BioAssay Systems, DTBA-100). This kit measures the concentration of thiobarbituric acid reactive substances (TBARS) that are formed as a result of lipid peroxidation. The assay is based on the reaction of TBARS with thiobarbituric acid to form a pink product. The absorbance at 535 nm is directly proportional to TBARS concentration in the sample. We similarly determined protein carbonyl content using a Protein Carbonyl Content Assay Kit (Sigma-Aldrich). Carbonyl content is determined by the derivatization of protein carbonyl groups with 2,4-dinitrophenylhydrazine (DNPH) leading to the formation of stable dinitrophenyl (DNP) hydrazone adducts, which can be detected spectrophotometrically at 375 nm, proportional to the carbonyls present. Finally, we used the EnzyChrom™ GSH/GSSG Assay Kit (BioAssay Systems) to determine total, reduced (GSH) and oxidized (GSSG) glutathione concentrations. The assay kit measures total glutathione activity using an enzymatic method in which Ellman’s Reagent (DTNB) reacts with glutathione to form a yellow product. The rate of change in absorbance at 412 nm is directly proportional to total glutathione concentration in the sample. To measure GSSG concentration, 1-methyl-2-vinylpyridinium triflate is first used to scavenge all GSH so that the remaining activity can be attributed to GSSG. The GSH concentration is then determined using the following equation: [GSH] = [glutahione_TOTAL_] − 2 × [GSSG]. Given the relatively small size of bumblebees and limited supernatant, all assays were performed in duplicate rather than triplicate.

### Statistical analysis

We analysed all data for statistical significance using RStudio (version 1.1.463) with R (version 3.6.1) and visualized the data using GraphPad Prism (v.9.0.0). To assess PPP activity, we used a three-factor analysis of variance (ANOVA) with a Geisser–Greenhouse correction to determine how the three fixed effects—experimental diet (^13^C_1_, 13C_2_), temperature (22°C, 32°C) and activity period (rest, exercise, recovery)—affect the δ^13^C signature of the breath. We similarly used a three-factor ANOVA to assess how experimental diet, temperature and activity period affect CO_2_ production (VCO_2_). We subsequently performed Tukey honestly significant difference (HSD) multiple comparison tests to elucidate differences among groups. Finally, two-way ANOVAs were used to assess voluntary activity, lipid peroxidation, protein carbonyl content, GSH and GSSG concentrations and the GSH:GSSG ratio between bumblebees provided with 25% sucrose or water at 22°C and 32°C. When a significant interaction between the experimental diet (water, 25% sucrose) and exposure temperature (22°C, 32°C) was detected, we performed a Tukey HSD test to elucidate differences between groups. All data were initially tested for normality and homogeneity of variance. Results were considered significant at *α* = 0.05.

## Results

### P‌PP activity and metabolic rate

**Figure 1:**
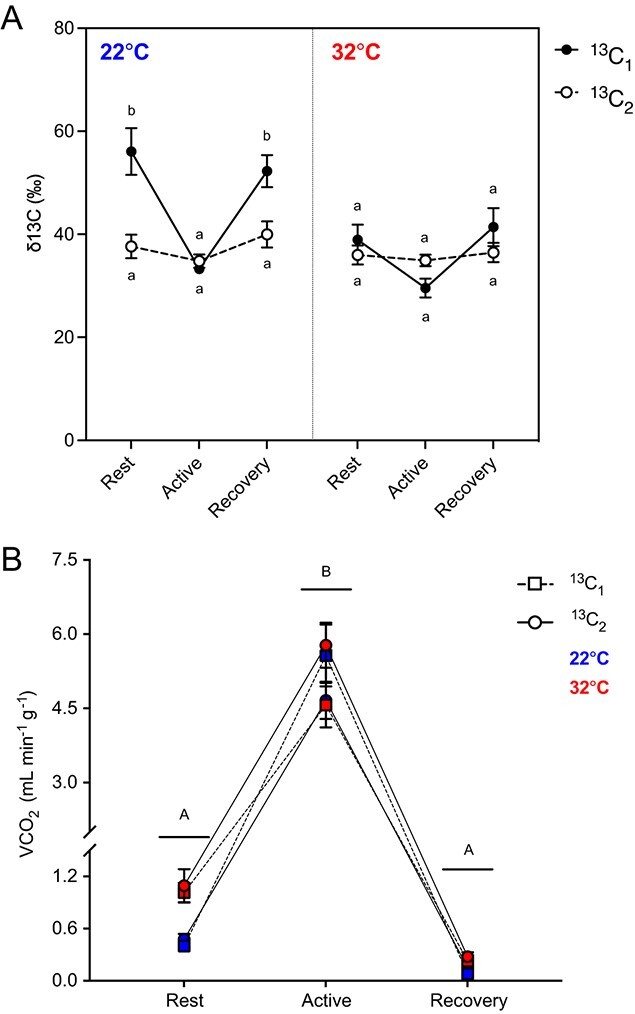
**(A)** The δ^13^C signature in the breath of *B. impatiens* fed a 25% sucrose solution enriched with isotopically labelled glucose on either carbon number 1 (^13^C_1_) or carbon number 2 (^13^C_2_) and exposed to 22°C or 32°C during rest, exercise and recovery periods. When δ^13^C values are higher for ^13^C_1_- than ^13^C_2_-fed bumblebees, it is indicative of PPP activity. Lowercase letters denote significant differences detected from pairwise comparisons between all groups. **(B)** The VCO_2_ of ^13^C_1_- and ^13^C_2_-fed bumblebees exposed to 22°C or 32°C during rest, exercise and recovery periods. Uppercase letters reflect the significant effect of the activity period on VCO_2_, determined from a pairwise comparison between all groups. Error bars represent the standard error. *n* = 7–8 per group.

In terms of PPP activity, we found a significant main effect of the experimental diet (three-factor ANOVA; *F*_1,75_ = 12.77, *P* = 0.001), temperature (three-factor ANOVA; *F*_1,75_ = 18.30, *P* < 0.001) and activity period (three-factor ANOVA; *F*_2,75_ = 17.12, *P* < 0.001) on the δ^13^C signature in the breath of *B. impatiens*. We also found a significant interaction between the experimental diet and temperature (three-factor ANOVA; *F*_1,75_ = 9.54, *P* = 0.003), as well as the experimental diet and the activity period (three-factor ANOVA; *F*_2,75_ = 9.16, *P* < 0.001). δ^13^C values were on average 33.1% higher in ^13^C_1_-fed bees during rest and recovery periods at 22°C compared to all other treatments (Tukey; *P* < 0.05) (Fig. 1A). We found a significant main effect of the activity period on the VCO_2_ of *B. impatiens* (three-factor ANOVA; *F*_2,72_ = 342.4, *P* < 0.001) and a significant interaction between the experimental diet, temperature and activity period (three-factor ANOVA; *F*_2,72_ = 4.29, *P* = 0.017; Fig. 1B). VCO_2_ was 11-fold higher in bees during exercise than during rest and recovery periods (Tukey; *P* < 0.05). Although we found no significant main effect of temperature on VCO_2_ (three-factor ANOVA; *F*_1,72_ = 2.59, *P* = 0.11), the VCO_2_ of bumblebees during rest and recovery periods was 59% and 56% higher at 32°C than 22°C, respectively (Fig. 1B).

### Voluntary walking activity

**Figure 2:**
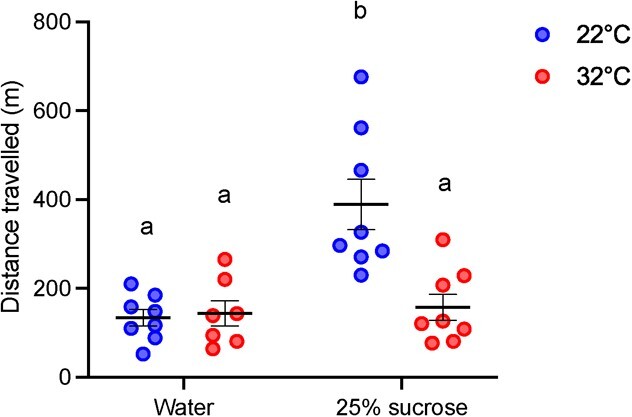
The total distance that *B. impatiens* provided with either water or a 25% sucrose solution travelled during a 5-hour exposure to 22°C or 32°C. Lowercase letters denote significant differences between experimental groups. Error bars represent the standard error. *n* = 7–8 per group.

During the 5-hour thermal exposure experiment, we found a significant interaction between the experimental diet and temperature on bumblebee walking activity (two-way ANOVA; *F*_1,27_ = 10.84, *P* = 0.003; Fig. 2). Bees exposed to 22°C and provided with 25% sucrose travelled 388.8 m (~78 m per hour) in 5 hours, whereas all other groups travelled 145 m (~29 m per hour) on average (Tukey; *P* < 0.05). We found no relationship between bee activity and any oxidative stress (lipid peroxidation, protein carbonyl) or antioxidant defence markers ([GSH], [GSSG] and GSH:GSSG) (Supplementary Fig. S2).

### Oxidative stress and antioxidant defence markers

**Figure 3:**
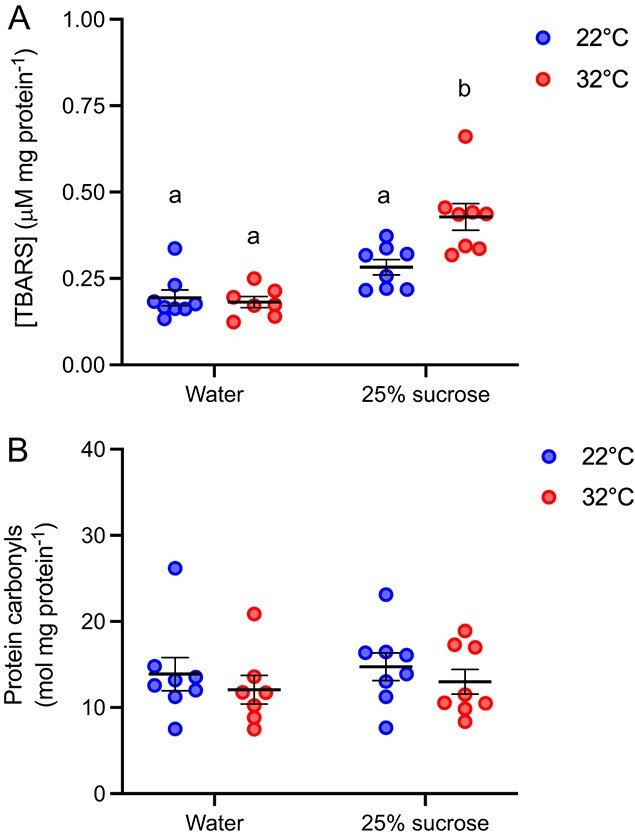
**(A)** The TBARS concentration and **(B)** protein carbonyl content in *B. impatiens* provided with either water or a 25% sucrose solution during a 5-hour exposure to 22°C or 32°C. Lowercase letters denote significant differences in the TBARS concentration between treatment groups. Error bars represent the standard error. *n* = 7–8 per group.

**Figure 4:**
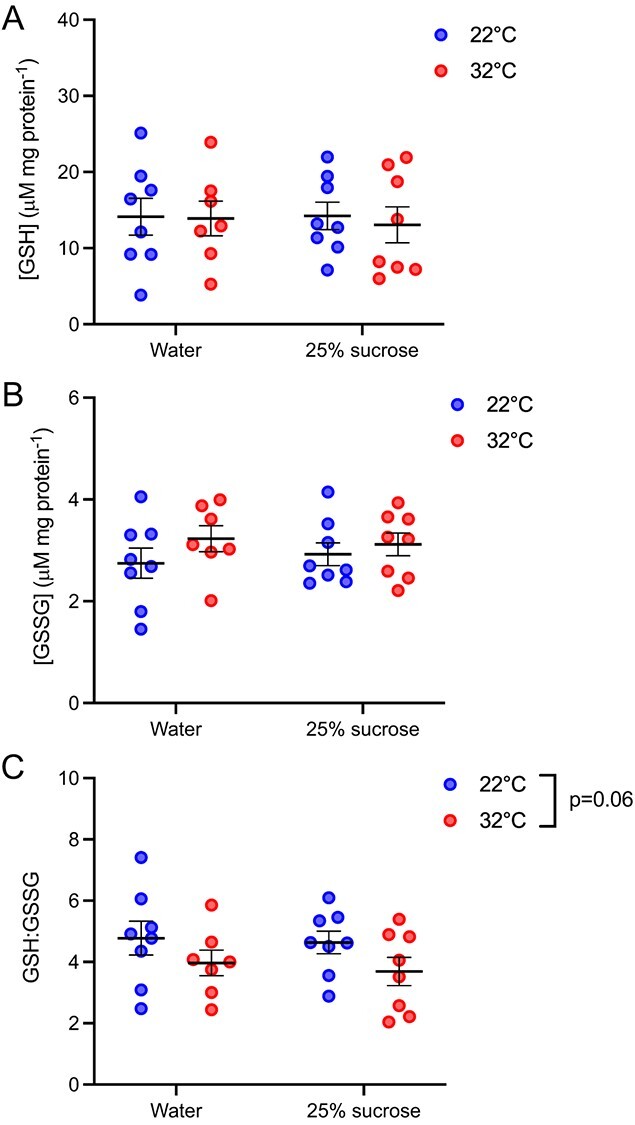
**(A)** The GSH concentration, **(B)** GSSG concentration and **(C)** GSH:GSSG ratio in *B. impatiens* after a 5-hour exposure to 22°C or 32°C and provided with either water or a 25% sucrose solution. Error bars represent the standard error. *n* = 7–8 per group.

We found a significant interaction between the experimental diet and temperature on the TBARS concentration in *B. impatiens* (two-way ANOVA; *F*_1,27_ = 8.76, *P* = 0.006) (Fig. 3A). The TBARS concentration in bumblebees provided with 25% sucrose and exposed to 32°C was 48.8% higher on average than all other treatment groups (Tukey; *P* < 0.05). We found no effect of neither the experimental diet (two-way ANOVA; *F*_1_,_27_ = 0.28, *P* = 0.68) nor the temperature (two-way ANOVA; *F*_1_,_27_ = 1.09, *P* = 0.31) on protein carbonyl content (Fig. 3B). Likewise, neither the experimental diet or the temperature had an effect on GSH (two-way ANOVA; diet, *F*_1,27_ = 0.03, *P* = 0.87; temperature, *F*_1,27_ = 0.11, *P* = 0.74) or GSSG concentration (two-way ANOVA; diet, *F*_1,27_ = 0.05, *P* = 0.83; temperature, *F*_1,27_ = 2.15, *P* = 0.15) in *B. impatiens* (Fig. 4A and B). However, we found a trend towards a lower GSH:GSSG ratio in bees exposed to 32°C (two-way ANOVA; *F*_1_,_27_ = 3,75, *P* = 0.06) but no effect of experimental diet (two-way ANOVA; *F*_1_,_27_ = 0.20, *P* = 0.66; Fig. 4C). The GSH:GSSG ratio in bees exposed to 22°C was 18.7% lower than those exposed to 32°C.

## Discussion

In support of our hypothesis, we found that heat exposure limits PPP activity in *B. impatiens.* At moderate summer temperatures (22°C), the δ^13^C signature was higher in bumblebees fed ^13^C_1_ than those fed ^13^C_2_ (when not exercising), indicating significant PPP activity. In contrast, the δ^13^C signature was similar between ^13^C_1_- and ^13^C_2_-fed bees during heat exposure (32°C), which is suggestive of a greater glucose flux through glycolysis rather than the PPP. We also found that exercise significantly reduced the relative flux of glucose through the PPP at 22°C, lending additional evidence for a trade-off between PPP activity and immediate ATP production when energetic demands are high. In general, bumblebees exhibited relatively little oxidative damage after a 5-hour thermal exposure, although lipid peroxidation was significantly higher in 32°C-exposed bees provided with 25% sucrose. We also found a slightly lower GSH:GSSG ratio in bees exposed to 32°C than 22°C, which is a suggestive of lower antioxidant potential. Overall, our findings indicate the PPP is routinely used by *B. impatiens* at moderate temperatures and that its activity is critically affected by acute heat exposure. Relatively short-term heat exposure (5 hours) did not result in significant oxidative damage but slightly reduced antioxidant potential. Our findings suggest that attenuated PPP activity in a warming climate may have some physiological consequences for *B. impatiens*, but it remains unclear how continued and more severe warming will impact this economically and ecologically important species.

### P‌PP activity and metabolic rate

We predicted that lower PPP activity at elevated temperatures may be the result of higher ATP demands. Indeed, we found that the VCO_2_ of bumblebees during rest and recovery periods was 59% and 56% higher at 32°C than 22°C, respectively. Interestingly, we found no evidence for a temperature-dependent change in VCO_2_ in bumblebees during exercise, as the average VCO_2_ values at 22°C and 32°C were 5.15 ± 0.39 and 5.21 ± 0.35 ml min^−1^ g^−1^, respectively. Previous studies have demonstrated that bumblebees are heterothermic, meaning that they can transition between endothermic and ectothermic states (Heinrich, 1975, 1976). In preparation for flight, bees use endogenous heat production to raise their thoracic temperature to an adequate temperature for muscle performance, usually between 30°C and 40°C (Heinrich, 1975; Heinrich and Esch, 1994). Thus, the bumblebees in our study likely exhibited similar thoracic temperatures and therefore similar metabolic demands during exercise despite exposure to different thermal regimes (Glass and Harrison, 2022). Accordingly, we found no differences in PPP activity in 22°C- and 32°C-exposed bees during the exercise period. During the exercise period, however, bees at 22°C exhibited a significant decline in PPP activity compared to when at rest, presumably owing to the energetic costs associated with raising thoracic temperature in the pre-flight warm-up, as well as the exercise bout itself. These findings are consistent with those in hawkmoths (*M. sexta*), which were found to shunt recently ingested nectar glucose to the PPP except during periods of exercise (Levin *et al.,* 2017). During exercise, δ^13^C_1_ was reduced by approximately 50% in hawkmoths and 42% in our 22°C-exposed bumblebees, suggesting that supplying muscles with ATP during flight may outweigh the need to produce NADPH, which can help generate antioxidant potential. Interestingly, hawkmoths exhibited an increase in δ^13^C_2_ during exercise, likely reflecting enhanced glycolysis and citric acid cycle activity. During glycolysis, all six carbons in the glucose ring (C_1–6_), including C_2_, are used to form acetyl-CoA. Acetyl-CoA is then used as a substrate in the citric acid cycle, resulting in the exhalation of all six carbons as CO_2_ in the breath (Levin *et al.,* 2017). We found no such increase in δ^13^C_2_ in *B. impatiens*, possibly because the ability to fuel exercise with recently ingested glucose and/or alternative metabolic fuels differs between these species. Indeed, bumblebees have been found to use trehalose (Surholt *et al.,* 1988; Stabler *et al.,* 2015; Theodorou *et al.,* 2022), proline (Teulier *et al.,* 2016; Stec *et al.,* 2021) and fructose metabolites (Newsholme *et al.,* 1972; Staples *et al.,* 2004) to fuel flight—metabolic fuels that would not be isotopically labelled using our protocol. Taken together, our study demonstrates that acute heat stress and brief periods of exercise can limit PPP activity in *B. impatiens*. As our climate continues to warm, studies that uncover how chronic/repeated heat exposure impacts the partitioning of dietary glucose across different metabolic pathways may be of great importance.

### Oxidative stress and antioxidant defences

We expected that the lower PPP activity exhibited by *B. impatiens* at elevated temperatures would result in oxidative damage and lower antioxidant potential. We tested this idea by exposing bees to a similar thermal regime (i.e. 22°C or 32°C for 5 hours) as in the isotopically labelled glucose experiment discussed above, and then assessing oxidative stress and antioxidant defence markers. It is important to note that bees exhibited different modes of locomotion across these two experiments (i.e. acute flying bout versus walking) because of differences in experimental container size, which introduces a confounding factor to our study. However, we found a robust temperature-related PPP response in the isotopically labelled glucose experiment, such that PPP activity was markedly lower at elevated temperatures. We feel that it is reasonable to assume that the same thermal exposures would have similar effects on PPP activity, despite occurring in different-sized containers. Furthermore, it is well-established that flying is a far more energetically expensive form of locomotion than walking (Schmidt-Nielsen, 1972), and thus it is unlikely that PPP activity would be attenuated to the same extent in walking versus flying bees, although the duration of activity (i.e. 3 min of flying versus 5 h of walking) is an important consideration. Taken together, our study makes the critical assumption that temperature is the most important factor in the thermal exposure experiment, and that its effects on PPP activity parallel those seen in the isotopically labelled glucose experiment. Further work is required to better understand how the duration and intensity of exercise may alter thermal PPP responses in this species.

In terms of oxidative stress, we found no evidence for protein damage after a 5-hour exposure to 32°C. Greater lipid peroxidation occurred in bees that were provided with a 25% sucrose solution during the 32°C exposure, but not those that were provided with water. A number of studies have similarly demonstrated that heat stress can cause lipid peroxidation in insect tissues (Jia *et al.,* 2011; Zhang *et al.,* 2014; Miao *et al.,* 2020). For example, sycamore lace bugs (*Corythucha ciliata) exhibited increasing* malondialdehyde (MDA; marker of lipid peroxidation) *concentrations with increasing temperatures in both laboratory and field settings after only 2 hours (*Ju *et al.,* 2014*).* Relatively few studies have explored how food availability impacts lipid peroxidation in insects (e.g. McCue *et al.,* 2015), but several have shown that access to food can improve the ability of bumblebees to cope with a thermal insult more broadly (Vanderplanck *et al.,* 2019; Maia-Silva *et al.,* 2021). Notably, Quinlan *et al.* (2023) found that the survival rate of *B. impatiens* exposed to 36°C for 5 hours was significantly higher in fed versus starved bees. Access to food may provide the energy required to shunt heat from the thorax to the abdomen and/or to achieve evaporative cooling through the spiracles or mouth (Heinrich, 1976; Heinrich and Buchmann, 1986). In our study, the higher [TBARS] in hot, sugar-fed bees relative to those provided with water is somewhat contradictory to these previous reports. One possible explanation is that elevated metabolic rates associated with the digestion of sugar-water led to increased ROS production and therefore lipid damage, but this hypothesis warrants further investigation. Finally, although food availability was shown to improve heat tolerance in other bumblebee studies, elevated environmental temperatures can limit food availability by reducing foraging activity and floral nectar production (Hemberger *et al.,* 2022; White and Dillon, 2023). Indeed, our findings show that voluntary walking activity was significantly attenuated at elevated temperatures even when bees were provided access to sugar water. This paradox between the need for food to better cope with a thermal insult and limited food availability at elevated temperatures highlights the susceptibility of wild bumblebees to thermal stress.

We found a slightly lower GSH:GSSG ratio in 32°C-exposed bees, which lends some support to the idea that attenuated PPP activity at elevated temperatures lowers antioxidant potential. The GSH:GSSG ratio is a common proxy for antioxidant potential, with lower ratios indicating higher levels of oxidative stress (Rojas and Leopold, 1996; Korsloot *et al.,* 2004). GSH:GSSG ratios typically decline when GSH has been oxidized to GSSG during peroxidase disposal by glutathione peroxidase and/or when GSSG has not yet been recycled back to GSH by glutathione reductase (Rojas and Leopold, 1996; Korsloot *et al.,* 2004; Vašková *et al.,* 2023). A major role of the PPP is to generate NADPH that can be used by glutathione reductase as an electron donor to reduce GSSG back to GSH (Kidd, 1997; Stincone *et al.,* 2015), albeit NADPH generated from the PPP can also function as reducing power for anabolic process such as fatty acid synthesis and cholesterol synthesis (Levin *et al.,* 2017). Thus, it is possible that the lower GSH:GSSG ratio in 32°C-exposed bees may be a consequence of reduced PPP activity limiting NADPH availability and constraining the recycling of GSH. Our findings are consistent with those of Levin *et al.* (2017), in which hawkmoths also exhibited a lower GSH:GSSG ratio when PPP activity was manipulated by denying moths access to nectar glucose. Numerous other studies have similarly shown that thermal stress (both hot and cold) can reduce the GSH:GSSG ratio in insects (e.g. Lalouette *et al.,* 2011). Finally, the lower GSH:GSSG ratio in 32°C-exposed bees may explain why we found little evidence of oxidative damage in *B. impatiens*. Protein carbonyl formation and lipid peroxidation caused by thermal stress may have been mitigated by the activities of GSH, but this hypothesis warrants further investigation because there are a number of other antioxidant defences at play that were not assessed in our study. Notably, bees have been shown to engage a number of other antioxidant defences in response to elevated temperatures, including the upregulation of important antioxidant enzymes (e.g. superoxide dismutase, glutathione *S*-transferase, catalase) and genes involved with antioxidant defence mechanisms (e.g. thioredoxin) (Choi *et al.,* 2006; Orčić *et al.,* 2017; Li *et al.,* 2019; Li *et al.,* 2020).

## Conclusion

As global climate change continues to increase the prevalence and severity of extreme heat events, understanding the physiological mechanisms that cause species to be vulnerable to heat stress is of utmost importance. Here, we show that heat exposure limits PPP activity in an important North American pollinator, which may hinder their ability to combat oxidative stress by impairing the recycling of GSH, a critical antioxidant. Our experiments were performed over relatively short timescales (~5 hours), but longer, repeated and/or more severe heat exposures may result in more substantial physiological consequences for life-history characteristics, such as growth and longevity. Indeed, a primary cause of ageing is the accumulation of oxidative damage in cells from both intrinsic (e.g. oxidative phosphorylation) and extrinsic (e.g. UV radiation) sources (for a review, see Liguori *et al.,* 2018). If PPP activity is routinely suppressed in a warming world, will it accelerate the onset of ageing in *B. impatiens*? Similarly, the PPP produces ribulose-5-phosphate, a precursor for nucleotide and amino acid biosynthesis (Stincone *et al.,* 2015) that has been implicated in accelerating cell growth (e.g. Jiang *et al.,* 2014).

If PPP activity is routinely suppressed in a warming world, will it suppress growth? Overall, understanding the intricate balance between energy demands and the PPP’s role in antioxidant defence and nucleotide/amino acid synthesis is critical, especially in predicting long-term impacts of climate change to bumblebee populations across the globe.

## Supplementary Material

Web_Material_coae031
